# JNK inhibitor and ferroptosis modulator as possible therapeutic modalities in Alzheimer disease (AD)

**DOI:** 10.1038/s41598-024-73596-1

**Published:** 2024-10-07

**Authors:** Sherin Zakaria, Nashwa Ibrahim, Walied Abdo, Alaa E. El-Sisi

**Affiliations:** 1 Department of Pharmacology and Toxicology, Faculty of Pharmacy, Kafrelsheikh University, Kafrelsheikh, 33516 Egypt; 2 Department of Pathology, Faculty of Veterinary Medicine, Kafrelsheikh University, Kafrelsheikh, 33516 Egypt; 3 Department of Pharmacology and Toxicology, Faculty of Pharmacy, Tanta University, Tanta, 31512 Egypt

**Keywords:** Molecular biology, Neuroscience

## Abstract

Alzheimer disease (AD) is among the most prevalent neurodegenerative diseases globally, marked by cognitive and behavioral disruptions. Ferroptosis is a form of controlled cell death characterized by intracellular iron accumulation associated with lipid peroxide formation, which subsequently promotes AD initiation and progression. We hypothesized that targeting the ferroptosis pathway may help in AD management. Therefore, our study aimed to evaluate the potential neuroprotective effect of the antifungal Ciclopirox olamine (CPX-O) that acts through iron chelation. We employed CPX-O separately or in combination with the JNK inhibitor (SP600125) in a mice model of AlCl_3_-induced AD. Animals underwent examination for behavioral, biochemical, histological, and immunohistochemical findings. Our results revealed that AlCl_3_ was associated with disruptions in learning and memory parameters, neuronal degeneration in the hippocampus, increased immunoreactivity of amyloid-β and tau proteins, a significant rise in iron, nitric oxide (NO), malondialdehyde (MDA), JNK, and P53 levels, along with the significant decrease in glutathione peroxidase activity. Interestingly, the administration of CPX-O alone or in combination with SP600125 in the AlCl_3_-induced AD model caused an improvement in the previously described examination findings. Therefore, CPX-O may be a promising candidate for AD treatment, and future clinical trials will be required to confirm these preclinical findings.

**Supplementary Information:**

The online version contains supplementary material available at 10.1038/s41598-024-73596-1.

## Introduction

Alzheimer disease is a dementia-associated disorder characterized by progressive neurodegenerative features, primarily within the cortical and hippocampal neuronal areas^1^. Recently, approximately 50 million individuals worldwide have suffered from AD. Unfortunately, this number is anticipated to triple by the year 2050, with a higher incidence expected in low- and middle-income countries^2,3^. Unfortunately, current FDA-approved therapies for AD only alleviate symptoms and do not provide a cure for AD^4^. Therefore, there is an urgent need for more research on other effective agents in AD treatment, especially in the early stages^5^. As of now, the precise mechanism of AD remains unknown. However, several predisposing factors have been associated with the development of AD, including genetic predisposition, neuroinflammation, dysbiosis of the gut microbiota, certain vascular-related diseases such as hypertension and atherosclerosis, and other disturbances related to oxidative injury, such as iron dyshomeostasis^1^.

Zhao et al. and Van Bergen et al. reported that neural iron accumulation accelerates the conversion of beta-amyloid (Aβ) fibrils into β-amyloid plaques with the further deposition of neurofibrillary tangles (NFTs) through the hyperphosphorylation of tau protein^6,7^. The formed β-amyloid plaques and NFTs are considered the main pathological features of AD^5^.

Ferroptosis is a condition associated with increased intracellular iron accumulation, leading to subsequent oxidative stress-related cellular death^8,9^. The cell death scenario in the ferroptotic pathway is based on three determined steps. The first involves the depletion of the cysteine glutamate antiporter system (Xc-system)/redox glutathione/glutathione peroxidase 4 (GPX4). The second is an increase in intracellular free iron, and the third is the oxidation of membrane Polyunsaturated Fatty Acids (PUFAs). Therefore, inhibiting the Xc-system and GPX4 are suggested as potential triggers for the initiation of ferroptosis^10,11^. It is noteworthy that iron is an essential redox metal for many physiological processes. Thus, the disturbance of the iron homeostatic balance poses a significant obstacle to normal cellular functions. The brain is highly sensitive to any abnormal elevation of iron levels, resulting in the oxidation of lipids and proteins in neurons, ultimately leading to neurotoxicity^12^. The fundamental pathway of iron metabolism involves iron circulating in the form of ferric iron (Fe^3+^), binding to transferrin, being imported into cells after forming a complex with transferrin receptor 1 (TFR1), and entering the cell through cell endosomes. Once inside, it is reduced to Fe^2+^ by the iron reductase enzyme and stored within the cells as ferritin^13^. Ferroptosis is primarily caused by excessive intracellular Fe^2+^, which oxidizes cellular lipids through a reaction known as the Fenton reaction. In this process, Fe^2+^ reacts with H_2_O_2_, generating the hydroxyl radical (^•^OH), considered the primary oxidative oxygen species in ferroptosis associated with the peroxidation of cellular lipids, proteins, and nucleic acids^14^.

Ferroptosis is also associated with different signaling pathways including mitogen-activated protein kinases (MAPKs)^15^. The MAPK family comprises extracellular signal-regulated kinase (ERK), p38, and JNK^16^. Wu and his colleagues demonstrated that ferroptosis induction is increased with upregulated expression of JNK protein^17^. Therefore, ferroptosis and JNK play a crucial role in AD pathogenesis. JNK can activate β- and γ-secretases, which are involved in the degradation of amyloid precursor proteins (APP), forming Aβ plaques^18^. Thus, phosphorylation of APP and tau proteins results in the deposition of both amyloid (Aβ) fibrils in senile plaques (SPs) and NFTs, respectively^19^. Based on the above information, we infer that tau protein hyperphosphorylation could be associated with excessive iron accumulation and JNK activation, or both. Everett et al. stated that amyloid plaques reduce Fe^3+^ to Fe^2+^, and the excess of Fe^2+^ enhances the Fenton reaction, generating hydroxyl radicals and contributing to ferroptosis-induced oxidative stress in AD^20^. The aforementioned data declared the relationship between JNK and iron accumulation. We suggest there is an indirect relationship between JNK and iron accumulation through Aβ plaque formation. Meanwhile, it is not clear whether targeting these active molecules may affect the progression of AD. Thus our hypothesis is focused on the possibility of the modulation of ferroptosis in combination with a JNK inhibitor may have a role in AD retardation. To validate this hypothesis we selected Ciclopirox olamine (CPX-O) as a ferroptotic modulator which is a synthetic antifungal hydroxypyridone derivative^21^, that acts through chelation of polyvalent metal cations in particular iron (Fe^3+^)^22^ and SP600125 as a competitive JNK inhibitor.

## Materials and methods

### Animals

The study was conducted on 30 male albino mice weighing 20–30 g. Mice were purchased from The Egyptian Organization for Biological Products and Vaccines (Agouza, Giza, Egypt). All animals were housed in well-ventilated plastic cages inside the animal house of the Faculty of Pharmacy, Kafrelsheikh University, Kafrelsheikh, Egypt. All mice were acclimatized for a week and kept under standard environmental conditions (22–25 °C temperature, 12 h light-dark cycle, 55 ± 5% relative humidity) during the experimental period. Both standardized pellet diet and water were *ad libitum*. All experimental procedures and methods were performed in accordance with the National Institutes of Health guidelines and regulations and were approved by the Ethics Committee Guidelines at Kafrelsheikh University, for the care of the experimental animals under the license number (KFS-IACUC/106/ 2023). All experimental procedures were done in compliance with the ARRIVE guidelines.

### Drugs and chemicals

ALCL_3_ was obtained from the Central Drug House (P) Ltd – CDH Co. (Vardhan House, 7/28, Ansari Rd, Daryaganj, New Delhi, India). Carboxymethyl cellulose (CMC) and Dimethyl Sulfoxide (DMSO) were purchased from EL-Gomhoria Company (EL Amyria, Cairo, Egypt). SP600125 was obtained from MedChemExpress Co. (1 Deer Park Dr, Suite Q, Monmouth Junction, NJ 08852, USA). Ciclopirox Olamine (CPX) was obtained from Sigma Chemical Co. (St. Louis, MO, USA).

### Induction of AD model

Alzheimer disease was induced in mice using ALCL_3_. The dose (17 mg/kg/day) was selected based on previous studies in rats^23,25^. To adjust for mice, the mouse equivalent dose (mg/kg) was calculated as double the rat dose^26^, resulting in a corrected dose of 34 mg/kg. Additionally, a pilot study was conducted, confirming the effectiveness of the chosen dose for inducing AD in mice (Fig. S1).

### Experimental design

**Fig. 1:**
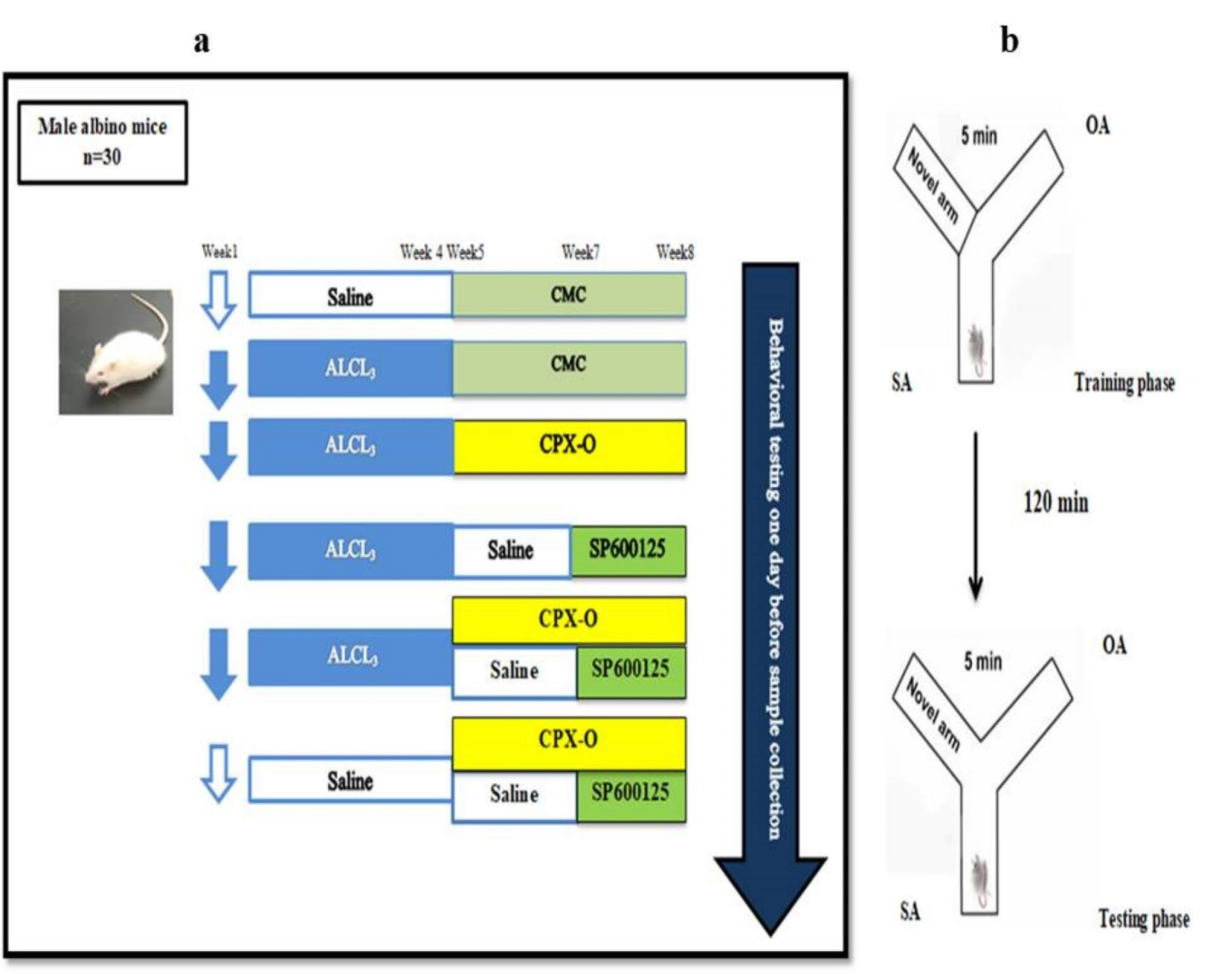
**(a)** Schematic Diagram of Experimental Design. Thirty mice were randomly classified into six groups (5 mice/group): Control (received saline), ALCL_3_ (orally gavaged with ALCL_3_ at 34 mg/kg daily for 4 weeks), ALCL_3_ + CPX-O, ALCL_3_ + SP600125, and ALCL_3_ + CPX-O + SP600125 (treated with ALCL_3_ followed by either CPX-O, SP600125 or their combination), respectively. The CPX-O + SP600125 group without ALCL_3_ represents the drug control group. ALCL_3_: Aluminum chloride, CPX-O: Ciclopirox olamine, CMC: carboxymethyl cellulose. (**b**) The cartoon figure represents the Y-maze task. Mouse photograph in this figure with an image was taken by the corresponding author in the laboratory at the Faculty of Pharmacy, Kafrelsheikh University.

Mice were distributed randomly into six groups (5 mice/ group) as illustrated in Fig. 1a. Five mice received only saline orally and served as the control group, and five animals were treated with both CPX-O and SP600125 and served as the **CPX-O + SP600125 group (sham group)**. Twenty mice were received ALCL_3_ for four weeks (34 mg/kg). At the beginning of the 5th week, mice were allocated into four groups. **ALCL**_**3**_ group: Mice were left as an ALCL_3_-induced AD model without any further treatment. **ALCL**_**3**_ **+ CPX-O** group: Mice treated with ALCL_3_ were given oral doses of CPX-O (20 mg/kg/day) for four weeks^27^. **ALCL**_**3**_ **+ SP600125** group: Mice treated with ALCL_3_ received SP600125 (16 mg/kg/day) intraperitoneally for two weeks starting from the 7th week^28^. **ALCL**_**3**_ **+ CPX-O + SP600125** group: Mice treated with ALCL_3_ received a combination of CPX-O and SP600125 as previously described.

### Behavioral testing

The Y-Maze test was used to measure mouse short-term memory^29^. Briefly, mice were acclimatized for two days in the behavioral testing room. The Y-maze apparatus was designed with three random arms: the novel arm (NA), the starting arm (SA), and the other arm (OA). The Y-maze test has two stages involving the training and testing stages, which are separated by 2 h, and each stage takes 5 min. The NA was blocked throughout the first training stage, and mice were only permitted to move freely through the SA and OA after entering the SA. The NA was opened during the second testing stage, allowing mice to move freely through all three arms. The data obtained were expressed as the percentage of time spent in the novel arm from the total trial time. Figure 1b.

### Tissue sampling

After behavioral testing and at the end of the experiment, mice were euthanized after using ketamine and xylazine (100 mg/kg and 10 mg/kg, respectively)^30^. The whole brain was dissected and rapidly harvested on an ice-cooled glass plate, thoroughly washed with isotonic saline, divided into two hemispheres and then we trimmed the brain tissue around the hippocampal area: The first hemisphere was used for the preparation of brain hippocampal homogenate. Hippocampal tissues were homogenized in phosphate-buffered saline PBS (10% w/v, pH 7.4) and then the homogenates were centrifuged at 4000 rpm for 15 min at 4 °C. The resultant supernatant was separated and kept at -80 °C for biochemical and western blot analysis. The second hemisphere, particularly the hippocampal area, was immediately immersed in 10% buffered formalin solution (pH 7.4) for histopathological and immunohistochemical (IHC) examinations.

### Histopathological and immunohistochemical examination

Regarding histopathology, the brain hippocampal tissues were fixed in a 10% buffered formalin saline solution (PH7.4). Brain hippocampal tissues were then washed in distilled water for 5 min, dehydrated with serial dilution of ethanol, and then cleared with xylene. Hippocampal tissues were immersed in soft paraffin wax at 56 °C, cast, and sectioned at 5 μm thickness. The brain hippocampal sections were stained with hematoxylin and eosin (H&E) stain^31^. The slides were examined and photographed using a light microscope connected to a digital camera (Leica-DFC camera, Germany). The immunohistochemical examination was performed as follows: hippocampal sections were dewaxed, and immersed in citrate buffer solution (0.05 M, pH 6.8) for antigen retrieval. The sections were immersed in 0.3% H_2_O_2_ for 5 min and then covered with protein block solution for 30 min. The sections were incubated with the following primary antibodies: beta-amyloid polyclonal antibody (Invitrogen, USA, Cat No 51-2700, 1:200 dilution), mouse monoclonal tau antibody (Invitrogen, USA, Cat No MA5-12808, 1: 300 dilution) and GFAP monoclonal antibody (Invitrogen, USA, MA5-11757, 1: 200 dilution). After an overnight incubation of the previous antibodies, the slides were rinsed in phosphate-buffered saline, then incubated with secondary antibodies, mouse monoclonal secondary antibody (Cat# K3468, EnVision+™ System Horseradish Peroxidase Labelled Polymer; Dako) for GFAP monoclonal antibody, Phospho-Tau (Thr217) Polyclonal Antibody (Invitrogen, USA, Cat **#** 44–744) and a goat anti-rabbit secondary Aβ antibody (Cat# K4003, EnVision+™ System Horseradish Peroxidase Labelled Polymer; Dako) for 30 min at room temperature. Slides were visualized with a DAB kit and eventually counterstained with Mayer’s hematoxylin. The staining labeling indices of the antibodies were presented as positive area percentages using Image J analysis software (NIH, USA)^32^.

### Biochemical measurements

**Iron level**: Iron level was assessed in the brain hippocampal homogenates using an iron colorimetric assay kit according to the manufacturer’s instructions (Biodiagnostic Company, Dokki, Giza, Egypt, Cat No. IR 15 10).

#### Antioxidant effect of the drug

Assessment of oxidative stress in brain hippocampal tissue homogenates of mice was carried out through measurement of nitric oxide (NO) level, malondialdehyde (MDA) level, and glutathione peroxidase activity (GPX).

NO activity in brain tissue was measured directly by colorimetric method using an NO assay kit according to the manufacturer protocol (Biodiagnostic Company, Giza, Egypt. Cat No. NO 25 33). This assay was performed according to the Greiss method in which nitrite forms nitrous acid diazotize sulphanilamide in an acidic medium, which further can be coupled with N-(1–naphthyl) ethylenediamine resulting in the formation of a bright reddish–purple color azo dye. The color intensity can be measured at 540 nm. It is directly proportional to NO concentration.

The lipid Peroxide Malondialdehyde (MDA) was measured according to the MDA assay kit in compliance with the manufacturer’s instructions (Biodiagnostic Company, Giza, Egypt. Cat No. MD 25 29). This assay is based on the reaction between thiobarbituric acid (TBA) and MDA in an acidic medium to form thiobarbituric acid, which is a pink reactive product proportional to the MDA level in the sample.

The activity of GPX in the brain was measured according to the manufacturer protocol of the Biodiagnostic Company kit, Dokki, Giza, Egypt, Cat No GR 25 24. The kit employs UV method procedures that detect the change in absorbance per minute over 3 min at a wavelength of 340 nm (A_340_ / min). The GPX activity in the sample is positively proportional to the rate of decrease in the A_340_.

**Beta-amyloid (Aβ) and P53 measurement**: The brain hippocampal tissue levels of Aβ 42 were measured using ELISA kits according to the manufacturer instructions (BioSource Company, San Diego, CA, USA) with a sensitivity level of 1.0 pg/ml. P53 level was assessed using ELISA kits according to the manufacturer’s instructions (RayBiotech Company, 3607 Parkway Lane, Suite 100, USA, Cat No. ELR-p53) with a sensitivity level of 0.32 ng/ml. The Aβ 42 and P53 concentrations were determined based on standard and reported as pg/mg and ng/mg protein, respectively.

### Western blot analysis for measurement of JNK

Brain hippocampal tissue samples were run on sodium dodecyl sulfate-polyacrylamide gel electrophoresis (SDS-PAGE gels) for JNK to separate total protein. After that, the protein was transferred to a polyvinylidene difluoride (PVDF) membrane as a solid support. Then the incubation of the membrane with blocking buffer was carried out at 37 ◦C for one hour. After that, the membrane was washed using Tris-Buffered Saline (TBS) tween and incubated with primary antibodies, including anti-JNK (1:2000) and anti-β-actin (1:1000) purchased from Thermo Fisher Scientific Company, Waltham, Massachusetts, USA, at 4 ◦C overnight. After incubation, the blot was rinsed 3–5 times for 5 min with TBST, and then HRP-conjugated secondary antibody (1:3000) was added to the membrane. After that, incubation was done in HRP-conjugated secondary solution against the blotted target protein for 1 h at room temperature. The membrane was then washed again as previously described. Western Bright enhanced chemiluminescence (ECL) was used to detect the bound antibodies. The level of JNK protein was calculated relative to that of β-actin on the Chemi Doc MP imager.

### Analysis of drugs synergy

The coefficient of drug interaction (CDI) was used for the analysis of the effects of drug combination**s**^33^. It was calculated as follows: CDI = AB/ (A×B), where AB is the ratio of the effect of the combined regimen group to the control group; A or B is the ratio of the effect of each single regimen group to the control group. Thus, CDI values < 1, = 1, and > 1 indicate that the drugs are synergistic, additive, and antagonistic, respectively.

### Statistical analysis

The data was presented as mean ± standard deviation (mean ± SD). For the statistical analysis of significant differences between groups, one-way analysis of variance (ANOVA) was used followed by Tukey’s Multiple Comparison Test. Results were considered significant at *p* ≤ 0.05. All statistical analysis was done using GraphPad Prism version 5.0 (GraphPad Prism Software Inc., San Diego, CA, USA).

## Results

### Y maze test

**Fig. 2:**
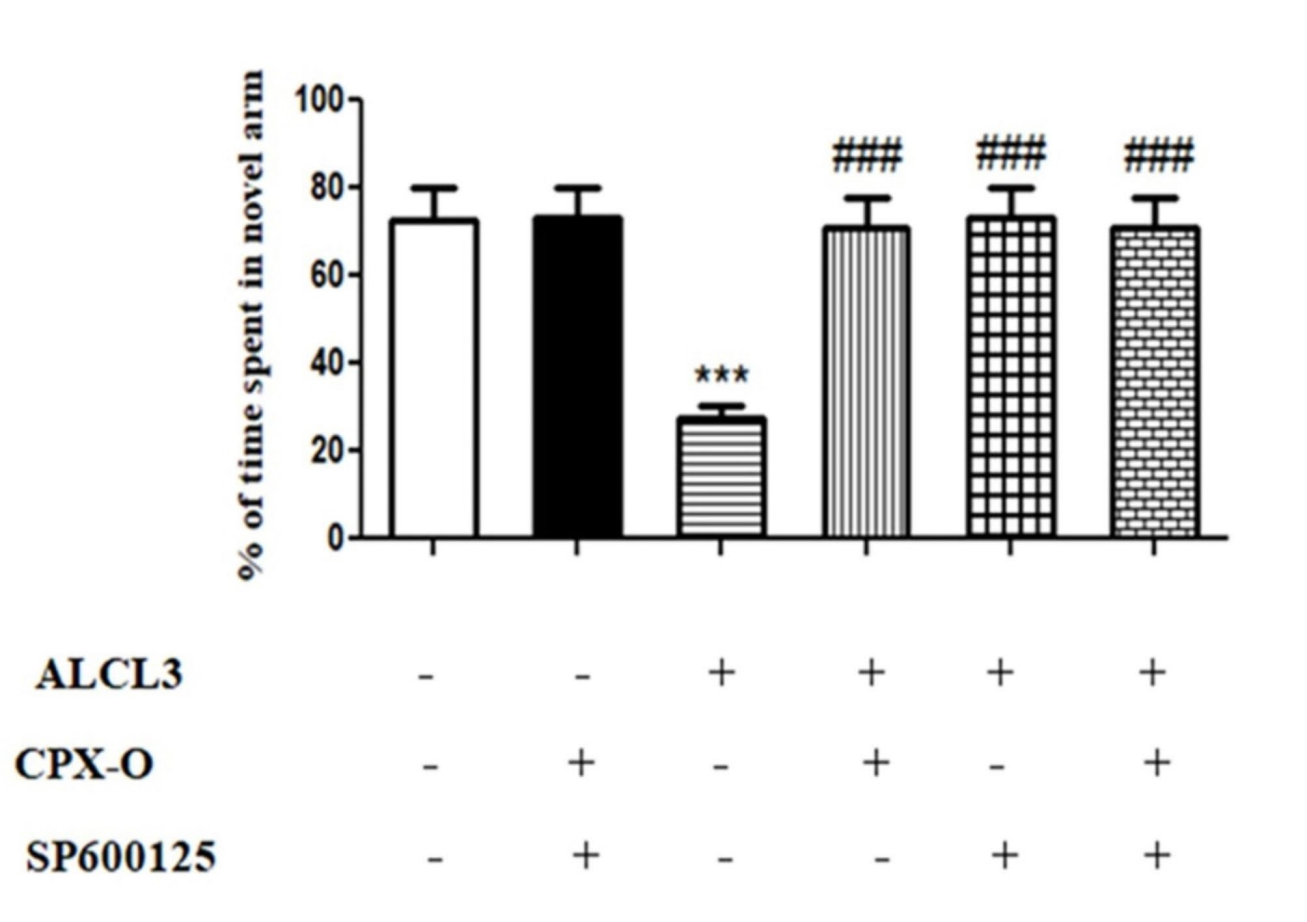
The effect of CPX-O and/or SP600125 on spatial working memory in the Y-maze behavioral test in AlCl_3_-induced AD in mice. CPX-O: Ciclopirox olamine. Data were expressed as means ± SD (*n* = 5). *** (*P* ≤ 0.001) Significant difference from the control, and ^###^ (*P* ≤ 0.001) significant difference from ALCL_3_.

Figure 2 represents the spatial working memory of the studied groups using the Y maze test. The observed data demonstrated a marked decrease in the percentage of time spent in the novel arm in the AlCl_3_ group than that observed for the control group (*p* ≤ 0.001). Diseased mice treated with CPX-O, SP600125, or their combination significantly increased the percentage of time spent in the novel arm compared to the AlCl_3_ group (*p* ≤ 0.001). Different treatment groups (CPX-O, SP600125, or their combination) showed non-significant differences between each other.

### Histopathological evaluation

**Fig. 3:**
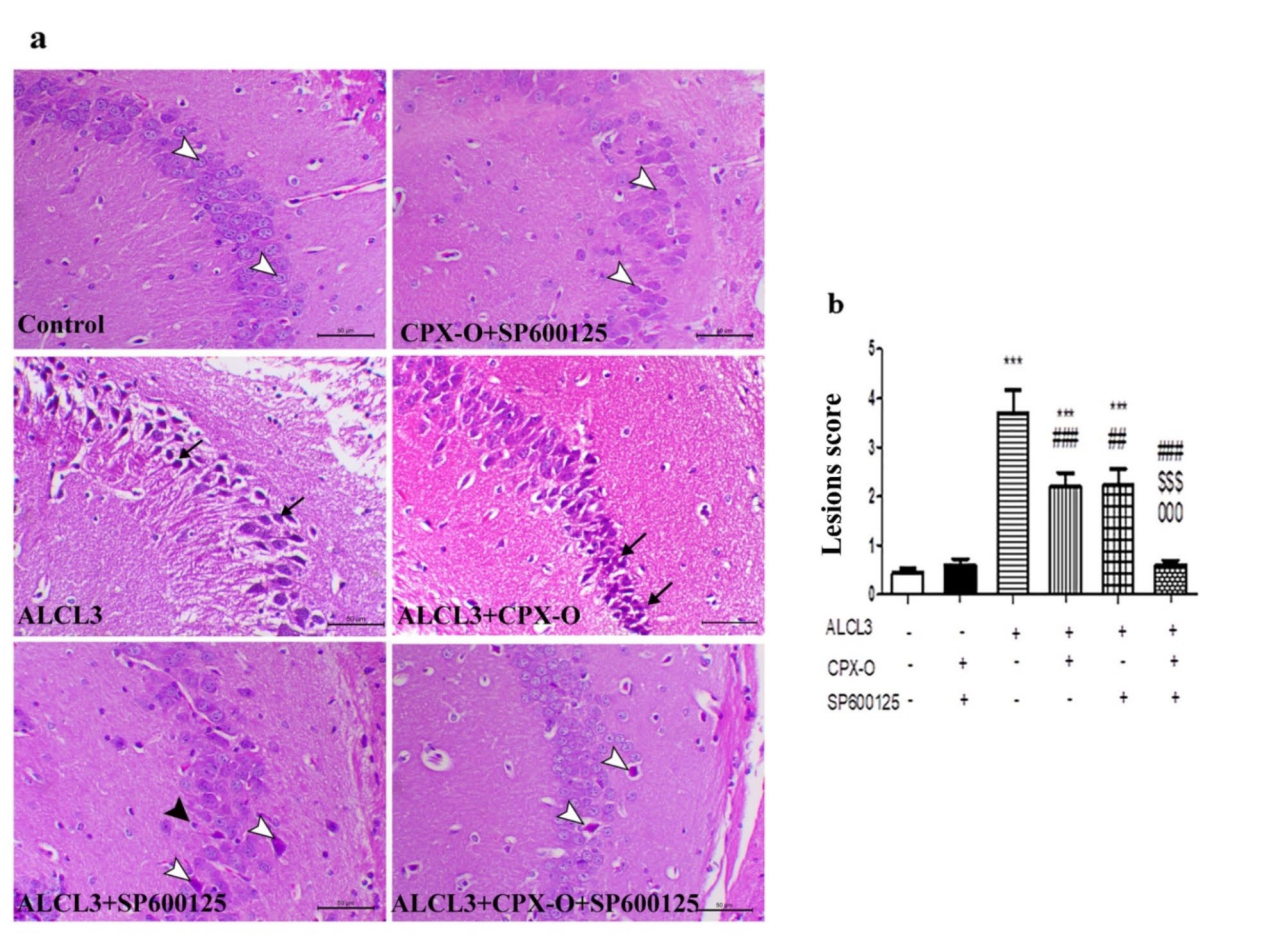
Histopathological findings of hippocampal sections in different studied groups (**a**) and quantitative lesion scores (**b**). The analysis of hippocampal sections in the control and drug control groups shows normal granular and molecular layers of CA2. The hippocampal section of the diseased group (ALCL_3_) exhibits features of severe ischemic neuronal degeneration associated with neuronal atrophy and nuclear pyknosis of the granular pyramidal neuronal cells (arrows). Treatment with CPX-O, SP600125, or their combination demonstrates a significant improvement in histological changes induced by ALCL_3_. White arrowheads indicate ischemic neurons. CPX-O: Ciclopirox olamine. The data are presented as means ± SD. *** (*P* ≤ 0.001) Significant difference from control. ^##^ (*P* ≤ 0.01), ^###^ (*P* ≤ 0.001) significant difference from ALCL_3_. ^$$$^ (*P* ≤ 0.001) significant difference from CPX-O. ^000^ (*P* ≤ 0.001) significant difference from SP600125.

Representative brain hippocampal sections stained with hematoxylin and eosin (H&E) are illustrated in Fig. 3a. Control and sham animals showed normal granular and molecular layers of CA2 in the hippocampus. On the other hand, the ALCL_3_-treated mice showed severe ischemic neuronal degeneration associated with neuronal atrophy, nuclear pyknosis of the granular pyramidal neuronal cells as well as increased microglia cell infiltration and remarkable neurophagia. The molecular layer revealed liquifactive changes associated with the disintegration of their fibers. CPX-O treated group showed a decrease in the atrophic degenerative ischemic changes within the pyramidal neuronal cells. SP600125 treated animals also showed multifocal ischemic neuronal injury within the granular neuronal cells. The combination group showed a marked decrease in the ischemic changes within the granular and molecular layers of the hippocampus.

Quantitative scoring of the pathological changes within the hippocampus demonstrated a marked increase in the lesion score within the ALCL_3_ group when compared to the control group (*P* ≤ 0.001). At the same time, CPX-O and SP600125 treated groups showed a significant decrease in the lesion score in comparison with the ALCL_3_ group (*P* ≤ 0.001). The combination group of CPX-O and SP600125 showed a marked decrease in the pathological lesions compared to the ALCL_3_-treated group, and its results went nearly toward the normal limits (Fig. 3b).

### Effect on hippocampal Aβ-amyloid protein level

**Fig. 4:**
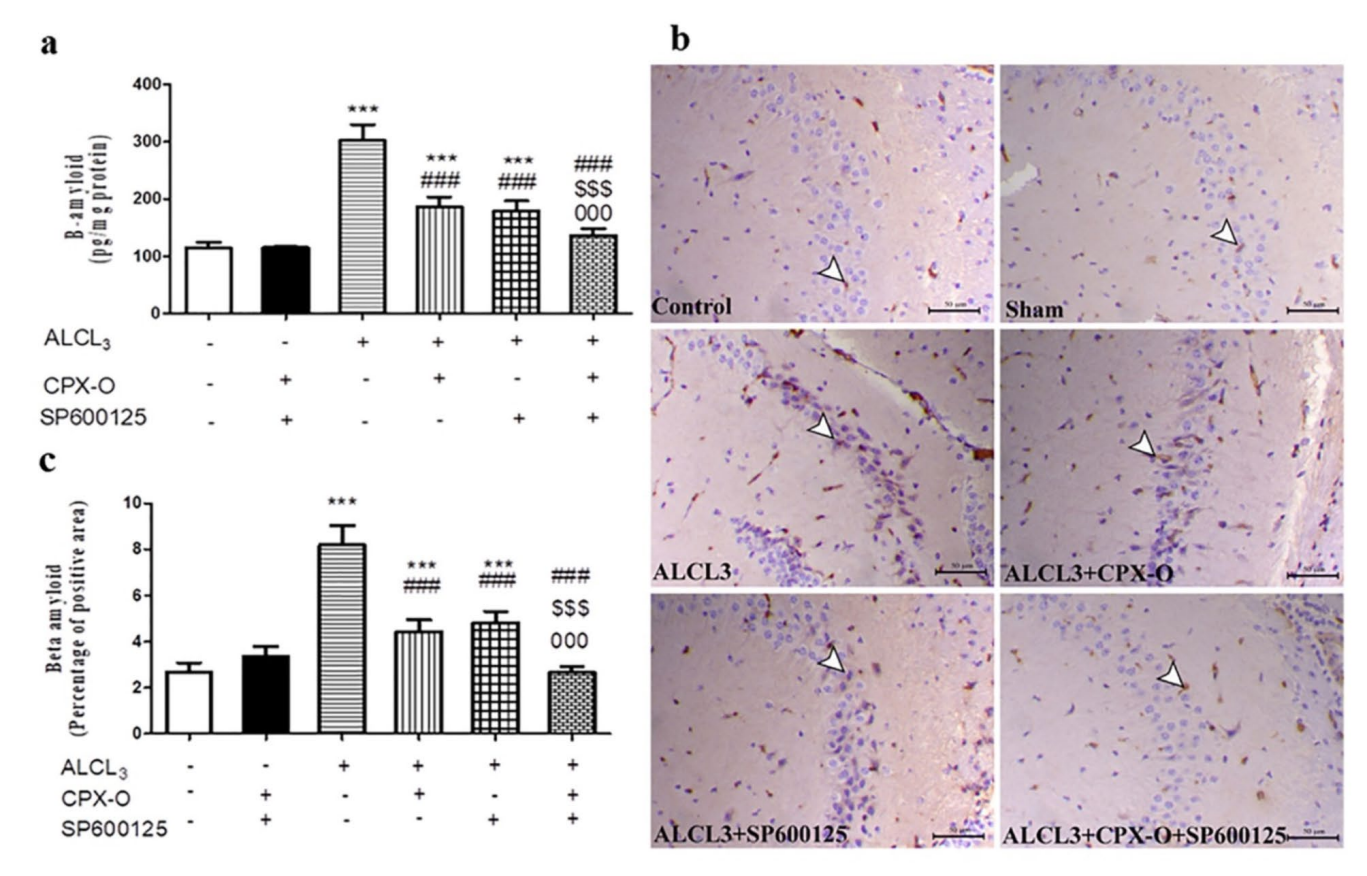
The effect of different treatment regimens on Aβ1–42 levels and immunoexpression. (**a**) Aβ levels (pg/mg protein) in different groups, (**b**) Microscopic images of brain hippocampal sections from the control, drug control or sham group (CPX-O and SP600125), ALCL3, CPX-O, SP600125, and combination groups immunostained with Aβ1–42 antibody. White arrowheads indicate the positively immunostained neuronal cells within the granular layer of the hippocampus. CPX-O: Ciclopirox olamine. (**c**) Quantitative scoring of the percentage of the positive area of Aβ1–42 antibody. Data are expressed as means ± SD (*n* = 5). *** (*P* ≤ 0.001) significant difference from control. ^###^ (*P* ≤ 0.001) Significant difference from ALCL_3_.,^$$$^ (*P* ≤ 0.01) Significant difference from CPX-O. ^000^ (*P* ≤ 0.001) significant difference from SP600125.

Our results revealed a significant elevation of β-amyloid level in ALCL_3_-treated mice by 163.76% in comparison with the control mice (*p* ≤ 0.001). Meanwhile, diseased mice treated with CPX-O, SP600125 alone or in combination significantly (*P* ≤ 0.001) dampened β-amyloid levels by 38.18%, 40.67%, and 55.07%, respectively compared to the ALCL_3_ treated mice (Fig. 4a).

### Immunohistochemical evaluation

ALCL_3_-treated mice showed a marked increase in Aβ plaques immunoexpression which was seen mostly within the prevascular and preneuronal areas. The expression of Aβ was markedly decreased within CPX-O, and SP600125 treated groups in comparison with ALCL_3_ (*p* ≤ 0.001). While the combination group significantly decreased the immunoexpression of Aβ nearly to the normal limits with significant differences when compared to CPX-O, and SP600125 groups (*p* ≤ 0.001). (Fig. 4b, c).

**Fig. 5:**
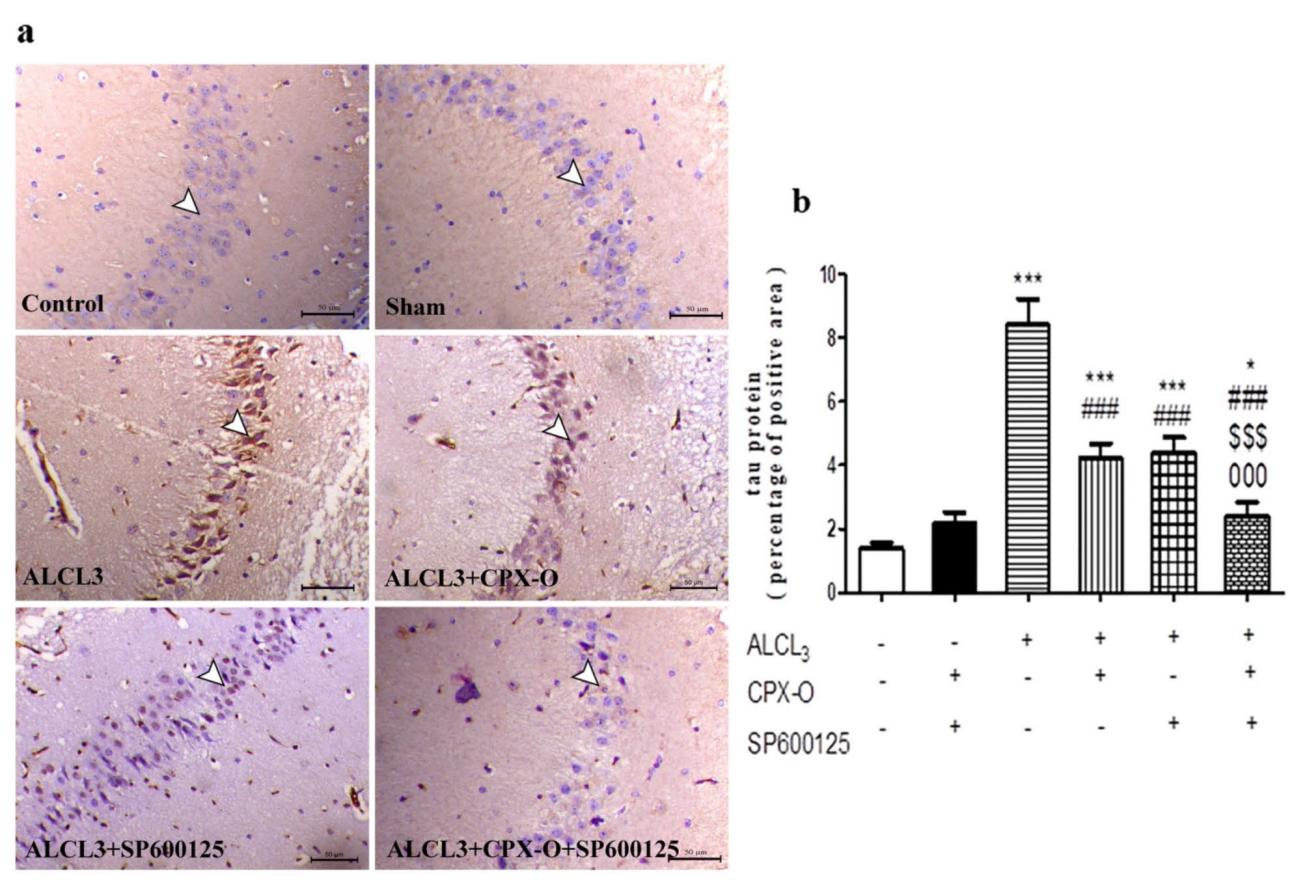
(**a**) Microscopic images of brain hippocampal sections from the control, drug control or sham group (CPX-O and SP600125), ALCL_3_, CPX-O, SP600125, and combination groups immunostained with tau antibody. White arrowheads indicate the positively immunostained neuronal cells within the granular layer of the hippocampus. CPX-O: Ciclopirox olamine. (**b**) Quantitative scoring of the percentage of the positive area of tau antibody. *( *P* ≤ 0.05), *** ( *P* ≤ 0.001) Significant difference from control. ^###^ ( *P* ≤ 0.001) Significant difference from ALCL_3_. ^$$$^ (*P* ≤ 0.001) Significant difference from CPX-O. ^000^ (*P* ≤ 0.001) Significant difference from SP600125.

The ALCL_3_ group revealed a marked elevation of immunoexpression of tau protein within the neuronal cells of the hippocampus in comparison with the control group (*p* ≤ 0.001). Contrary, diseased animals treated with CPX-O, and SP500125, showed a decrease in tau immunostaining in comparison with the ALCL_3_ group (*p* ≤ 0.001). The combination group revealed a marked decrease in tau expression compared to the ALCL_3_ group and other treatment groups (*p* ≤ 0.001). (Fig. 5a, b).

**Fig. 6:**
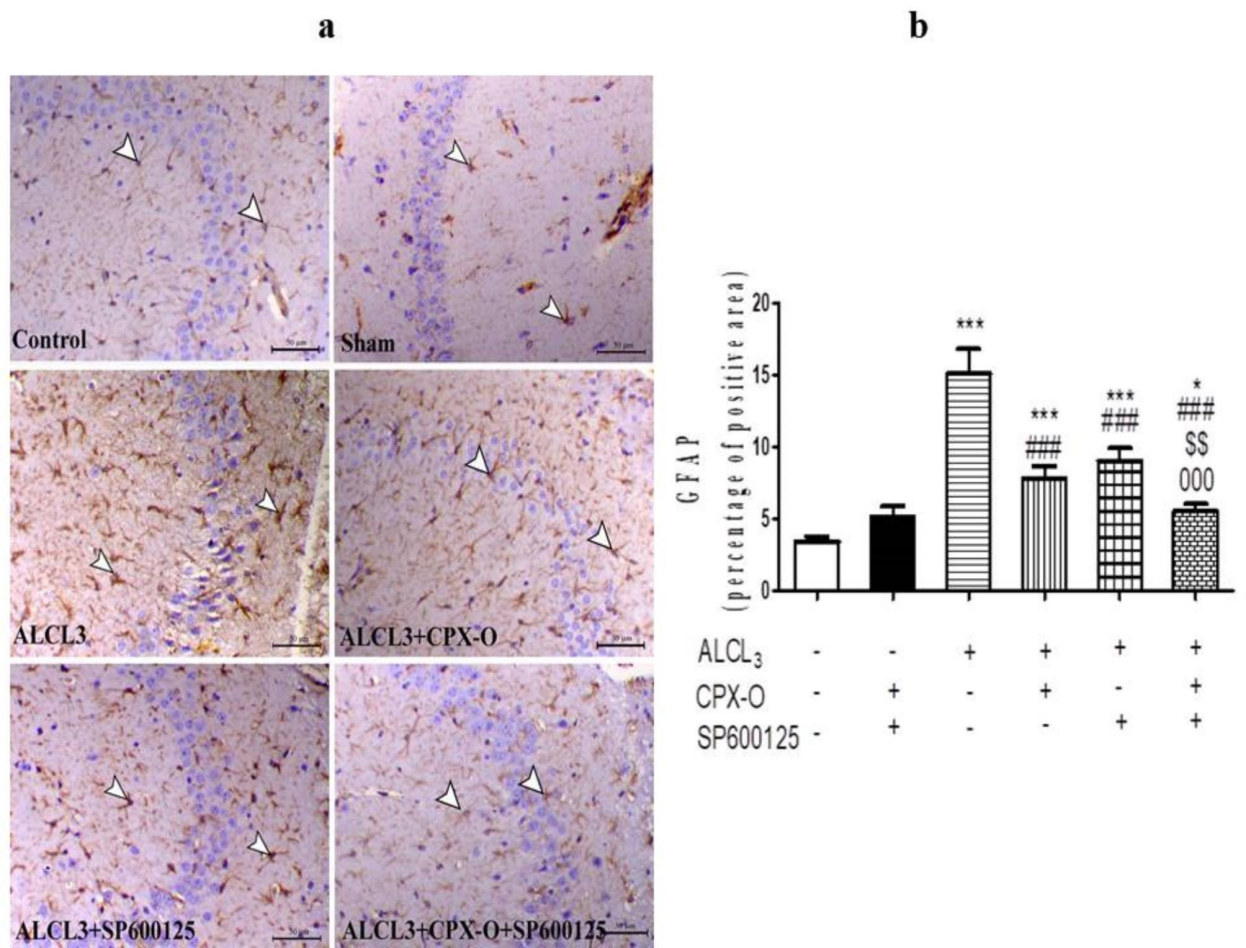
(**a**) Microscopic images of brain hippocampal sections from the control, drug control or sham group (CPX-O and SP600125), ALCL_3_, CPX-O, SP600125, and combination groups immunostained with GFAP antibody. White arrowheads indicate the positively immunostained astrocytic cells within the granular and molecular layers of the hippocampus. CPX-O: Ciclopirox olamine. (**b**) Quantitative scoring of the percentage of the positive area of GFAP antibody.*( *P* ≤ 0.05), *** ( *P* ≤ 0.001) Significant difference from control. ^###^ (*P* ≤ 0.001) Significant difference from ALCL_3_. ^$$^ (*P* ≤ 0.01) Significant difference from CPX-O. ^000^ (*P* ≤ 0.001) Significant difference from SP600125.

The ALCL_3_ group demonstrated a marked increase in the GFAP immunostaining associated with an increase in the number of astrocytes and even the thickness of their fibers compared to control and drug control groups (*p* ≤ 0.001). On the other hand, the expression of GFAP antibody was decreased within CPX-O and SP600125 groups compared to ALCL_3_ (*p* ≤ 0.001). Of note, the combination group showed a marked decrease in the expression of GFAP antibody within the hippocampus compared to the ALCL_3_ group (*p* ≤ 0.001), CPX-O (*p* ≤ 0.01), and SP600125 treated group (*p* ≤ 0.001) (Fig. 6a, b).

### Effect on brain iron level

**Fig. 7:**
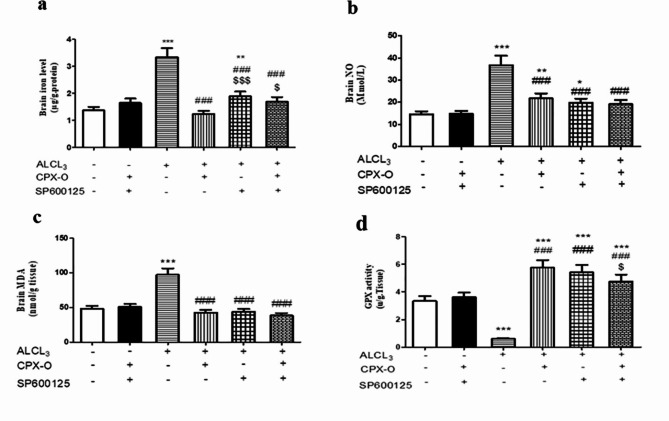
Effect of different treatment regimens on iron, NO, MDA levels, and GPX activity. (**a**): Brain tissue iron level (µg/g protein), (**b**): NO level (µmol/L), (**c**): MDA level (nmol/g tissue), and (**d**): Brain GPX activity (u/g tissue). Data are expressed as means ± SD (*n* = 5). *( *P* ≤ 0.05), **(*P* ≤ 0.01), *** ( *P* ≤ 0.001) Significant difference from control. ^###^ ( *P* ≤ 0.001) Significant difference from ALCL_3_. ^$^ (*P* ≤ 0.05), ^$$$^ ( *P* ≤ 0.001) Significant difference from CPX-O. CPX-O, ciclopirox olamine.

Iron level in the brain hippocampal tissue homogenate was markedly elevated in the ALCL_3_ group by 42.8% in comparison with the control group (*P* ≤ 0.001). In contrast, diseased mice treated with CPX-O, SP600125 alone, or in combination markedly decreased the iron brain level by 62.9%, 43.3%, and 49.6%, respectively in comparison with ALCL_3_ (*P* ≤ 0.001), with a remarkable decrease of its level to the normal limits in the combination group (Fig. 7a).

### **Antioxidant effect of the drugs**

ALCL_3_-treated mice showed a marked increase in NO brain levels by 152.6% compared to control mice (*P* ≤ 0.001). The NO level was markedly decreased by 40.93%, 46.3%, and 48.08% in CPX-O, SP600125, and their combination respectively compared to ALCL_3_-treated mice (*P* ≤ 0.001), with a remarkable decrease of its level to the normal limits in the combination group (Fig. 7b).

ALCL_3_-treated mice also showed a marked increase in their MDA level in hippocampal tissues by 101.9% in comparison with the control group (*P* ≤ 0.001). While the treated groups showed a decrease in the MDA level in the hippocampal by 56.2%, 54.9%, and 60.4% in CPX-O and SP600125 separately or in combination, respectively, in comparison with ALCL_3_-treated mice (*P* ≤ 0.001). Different treatment regimens (CPX-O, SP600125, or their combination) showed non-significant difference compared to the control and between each other (Fig. 7c).

Regarding GPX activity, the diseased mice showed a marked decrease in its activity by 82.41% relative to the control group (*P* ≤ 0.001). While the animals treated with CPX-O, and SP600125, or in combination significantly increased GPX activity by (879.5%, 820.3%, and 708.5%) respectively compared to ALCL_3_-treated mice (*P* ≤ 0.001). SP600125 treated mice showed a non-significant difference compared to CPX-O and combination groups, while the combination group showed a significant difference compared to CPX-O treated mice (*P* ≤ 0.05). (Fig. 7d).

### **Effect of drugs on JNK expression**

**Fig. 8:**
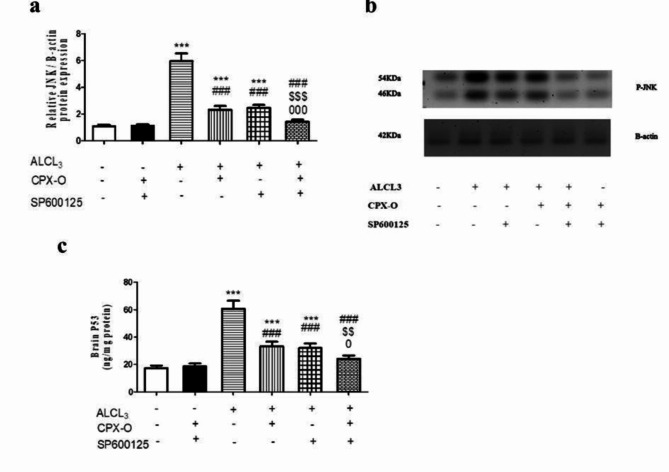
Effect of different treatment regimens on hippocampal JNK expression and P53 level. (**a**,** b)**: Protein expression of JNK/β-actin, and (**c)**: P53 level (ng/mg protein). Data are expressed as means ± SD (*n* = 5). *** ( *P* ≤ 0.001) Significant difference from control. ^###^ ( *P* ≤ 0.001) Significant difference from ALCL_3_. ^$$^ (*P* ≤ 0.01) ,^$$$^ (*P* ≤ 0.01) Significant difference from CPX-O. ^0^(*P* ≤ 0.05), ^000^ (*P* ≤ 0.001) Significant difference from SP600125.

The ALCL_3_ group exhibited a significant increase of JNK protein expression by 455.1%, in comparison with the control group (*P* ≤ 0.001). On the other hand, treatment with CPX-O, SP600125, and their combination revealed a significant decrease in JNK protein expression by 60.9%, 58.9%, and 76.4% respectively, in comparison with the ALCL_3_ treated mice (*P* ≤ 0.001). (Fig. 8a, b). Original blots are presented in Supplementary Figure S2.

### Effect on brain P53 level

Our results demonstrated a marked elevation of P53 level in the ALCl_3_ group in comparison with the control group by 247.5% (*P* ≤ 0.001). Contrary, the treatment of diseased mice with CPX-O, SP600125, and the combination of both drugs significantly decreased P53 levels by 45.1%, 47.2%, and 60.2%, respectively compared to the ALCL_3_ group (*P* ≤ 0.001). The combination showed a non-significant difference compared to control (*P* ≤ 0.05). (Fig. 8c)

## Discussion

There are still numerous challenges related to AD, concerning pathogenesis or treatment protocols. Among them are the absence of established underlying mechanism(s), the lack of specific treatment, and the continuous increase in morbidity and mortality rates^34^. Previous data have reported substantial evidence of the ferroptosis mechanism in the development of AD. Herein, we are attempting to target both iron accumulation and the JNK pathway, which may covalently participate in AD development, in a convenient modality to treat and/or retard the progression of AD. Previous data have stated that the administration of competitive inhibitors of JNK, such as SP600125, showed a great advantage in treating AD^35,36^. Meanwhile, SP600125 acts as an inhibitor of ferroptosis in neurodegenerative diseases (NDD)^37^. Thus, we hypothesize that the administration of CPX-O, which has a special privilege in iron chelation in fungal treatment, either alone or in combination with the JNK inhibitor SP600125, may be of great value in the management of AD through interference with ferroptosis events in the brain.

We performed several biochemical and behavioral measures to validate our hypothesis to assess AD in different treatment groups. Regarding the behavioral changes observed in the Y-maze test, our results revealed that co-administration of CPX-O and/or SP600125 either alone or in combination, may retard the progression of AD. It is noteworthy that the neuroprotective effect of SP600125 has been studied^38^, on the other hand, the CPX-O effect in the Alzheimer model is not reported before. Of note, the combination of both drugs showed a synergistic effect.

To further validate our hypothesis, we assessed the expression of Aβ and Tau proteins. The degree of neuronal loss in AD is markedly correlated with the deposition of Aβ plaques and hyperphosphorylation of tau protein^39^. Our results revealed a significant decrease in Aβ levels in brain tissue homogenates in mice treated with CPX-O, and SP600125 separately, or in combination. According to CDI, the combination regimen of both drugs demonstrated a high synergistic effect on decreasing Aβ levels. The aggregation of Aβ plaques has long been widely considered a crucial and first event in the development of AD^40^. The major component of these plaques is Aβ which is created by protease cleavage of APP^41^. APP can be broken down by the proteolytic action of secretases (α, β, and γ) to produce either amyloidogenic or non-amyloidogenic Aβ proteins. In the non amyloidogenic pathway, APP is cleaved by α-secretase resulting in the formation of the soluble amyloid precursor protein α (sAPP α)^42^. SAPPα is thought to have a neuroprotective effect^25^. While in amyloidogenic pathway, APP is cleaved by β-secretase leading to the formation of soluble amyloid precursor protein β (sAPβ) and C-terminal 99 fragment (CTFβ) and followed by γ-secretase cleavage to produce Aβ peptides. The aggregation of these peptides leads to the formation of oligomers, polymers, and eventually insoluble AB plaques^43^. However, an imbalance in the generation and clearance of Aβ can lead to its sedimentation^44^, which in turn can cause dysregulation of synapses and neurons^40^, ultimately playing a role in the pathophysiology of AD.In addition to Aβ formation, the presence of NFTs is considered a main pathological feature of AD^5^. These tangles are formed through abnormal hyperphosphorylation of tau protein forming insoluble fibers. Tau dissociates from microtubules and the fibers combine to form intracellular NFTs, which disrupt neural function and induce cell damage^45^.

The ameliorated effect of SP600125 could be explained by direct inhibition of the JNK pathway. This path is correlated with Aβ deposition through transcriptional and enzymatic activation of β- and γ-secretases, as well as phosphorylation and stabilization of APP pathway, and promotion of Aβ42 production, which is considered the main component of senile plaques^46,47^. It is unclear which event occurred first: JNK activation or Aβ accumulation, and what serves as the initiator for the other^48^. JNK activation also phosphorylates tau protein, dissociating it from the microtubules, ultimately leading to the formation of NFTs^49^.

We suggest that the effect of CPX-O on Aβ and tau protein accumulation is indirect through iron modulation. Neural iron accumulation accelerates the conversion of Aβ fibrils into plaques, with further deposition of NFTs through hyperphosphorylation of tau protein^6,7^.

Assessment of iron levels in the brain tissues confirmed this postulation. As shown in our results, CPX-O demonstrated a significant decrease of iron levels in AlCl_3_-treated mice. Moreover, SP600125 showed a significant decrease in iron levels in AlCl_3_-treated mice, implying a suggestive neuroprotective effect of these agents through the inhibition of ferroptosis. According to CDI analysis of drug synergy on iron levels, the combination of CPX-O and SP600125 showed a synergistic effect.

The molecular events associated with iron accumulation are highly correlated with neuronal damage where neuronal iron accumulation is mostly associated with the depletion of antioxidant enzymatic stores such as GSH and GPX enzymes, induction of the oxidative stress cascade, and a noticeable increase in lipid peroxides biomarkers such as NO and MDA in the brains of AD patients^50^. ROS plays a major role in ferroptosis-induced cell death and the further deterioration of AD^50,51^. It has been noted that ROS are derived from iron through the Fenton reaction in a sequential scenario in ferroptosis, which depends on GPX4. GPX4 is considered a negative regulator of ferroptosis through the reduction of ROS release and also by decreasing cellular iron uptake^52^. The depletion of GSH and subsequent GPX4 inactivation are crucial for inducing ferroptosis^53^. Therefore, ferroptosis could be partially or completely inhibited by antioxidant agents^8^. In the same way, our results revealed that the AlCl_3_-treated group showed a significant reduction in the activity of GPX and increased MDA and NO levels as a consequence of increased iron levels in the brain tissue homogenates, in contrast, CPX-O, SP600125, and their combination increased GPX activity and decreased MDA and NO levels. The effect of CPX-O on antioxidant activity is controversial depending on the model used. Previous studies have stated that the cytotoxic effect of CPX-O on colorectal and hepatocellular carcinoma is achieved through an increase in the intracellular levels of ROS^54,55^. Also, the fungicidal activity of CPX-O against different dermatophytes acts through an increased ROS level^56^. On the other hand, CPX-O showed a reduction in ROS levels in a dermatitis model in mice^57^. Also, CPX-O dampened oxidative stress-induced parthanatos^58^. In the same context, SP600125 ameliorated oxidative stress in streptozotocin-induced neurocognitive deficit in rats^59^. In addition, a previous study revealed that SP600125 has an anti-ferroptotic action by increasing the ratio of GSH/GSSG, decreasing tert-butylhydroperoxide (t-BHP)-induced production of lipid ROS, and restoring the activity of GPX4 in PC21 cells^37^. Intriguingly, Aβ deposition and oxidative stress act in a vice-versa manner, wherein Aβ promotes oxidative stress, and ROS stimulates Aβ deposition^60^ Notably, the anti-ferroptotic and, consequently, antioxidant effects of the therapeutic regimen used showed a synergistic effect. Fig S3.

It has been reported that Aβ deposition in the brain induces neuronal inflammation by activating microglia and astrocytes. Aβ has been found to stimulate the nuclear factor-kappa beta (NF-κB) pathway, one of the most significant regulators of pro-inflammatory gene expression in astrocytes, leading to an increase in complement C3 release. This, in turn, causes neuronal impairment and microglial activation^61^. The activated microglia lead to an increase in the levels of pro-inflammatory cytokines such as tumor necrosis factor-alpha (TNF-α), interleukin-1 (IL-1), IL-6, and IL-18. These cytokines contribute to neuronal death and tau hyperphosphorylation^62^. The process of astrocyte activation is associated with overexpression of some proteins, including glial fibrillary acidic protein (GFAP)^63^. GFAP serves as an intermediate filament protein in the astrocytic cytoskeleton. While its expression is typically low in healthy individuals, there is a significant increase in GFAP expression and concentrations observed in the brains of patients with AD, especially in regions surrounding Aβ plaques, and increased with the accumulation of tau in the brains^64,66^. These findings are consistent with our results, which demonstrated elevated immunohistochemical expression of GFAP in AlCl_3_-treated mice and its inhibition in different treated groups compared to the AlCl_3_ group.

Patients with AD exhibit a high level of p53 in several brain areas. Aβ accumulation is one of the leading factors accompanied by p53-dependent neuronal cell death^67^. Han et al. stated that Aβ triggers apoptosis through several ways including promotion of mitochondrial fission, disruption of mitochondrial membrane potential, elevating reactive oxygen species (ROS) levels, and activating mitophagy^68^. Another study carried out by Barrantes et al. also demonstrated that Aβ 42 induces DNA strand breaks, resulting in p53-mediated apoptosis^69^. P53 also contributes to AD through triggered stress response, and increased expression of Glycogen synthase kinase 3β (GSK3β) and phospho-tau^70^. Moreover, P53 is considered a positive regulator of ferroptosis^71^. P53 inhibits SLC7A11, a solute carrier in the Cystine/glutamate antiporter System xc−), inhibiting cystine exchange with glutamate^50^. This leads to a decrease in glutathione synthesis and consequent inactivation of the GPX enzyme, resulting in oxidative stress and the excessive accumulation of lipid peroxides, ultimately inducing ferroptosis^72^. In addition to SLC7A11, P53 can affect solute carrier family 25 member 28 (SLC25A28) in mitochondria^73^. Activated P53 can stimulate the activity of SLC25A28, leading to the abnormal assembly of redox-active iron and enhancing ferroptosis^74^. P53 also facilitates the entry of Fe^2+^ into cells by up-regulating the expression of TFR1^75^. However, the exact effects and underlying mechanisms of these actions remain to be clarified. P53 can affect lipid peroxidation by targeting spermidine/spermine N1-acetyltransferase 1 (SAT1) and enhancing its expression. This elevation in SAT1 levels increases arachidonic acid 15-lipoxygenase-1 (ALOX15). Subsequently, ALOX15 promotes lipid peroxidation, inducing ferroptosis^76^. Moreover, numerous p53 target genes have been identified that can facilitate cell ferroptosis, one of them is glutaminase 2 (GLS2). P53 activation directly interacts with the promoter region of GLS2, facilitating the transcription of GLS2. GLS2 can enzymatically break down glutamine into glutamate, thereby diminishing antioxidant capacity by reducing GSH levels. This reduction in GSH levels enhances sensitivity to ferroptosis^77^. This is consistent with our results, which referred to the antiferroptotic effect associated with the dampened P53 levels induced by the JNK inhibitor SP600125 and the iron chelator CPX-O.

## Conclusion

The inhibition of ferroptosis by CPX-O, an iron chelator, and SP600125, a JNK-dependent signaling pathway modulator, is a promising therapeutic strategy in AD. The interventions employed (CPX-O and SP600125) demonstrated a synergistic effect in retarding neurodegeneration in AD induced by AlCl_3_ in mice. This therapeutic combination ameliorated Aβ accumulation, possibly through ferroptosis-correlated molecular events, such as decreasing oxidative stress, GFAP expression, and apoptosis.

## Electronic supplementary material

Below is the link to the electronic supplementary material.

Supplementary Material 1

## Data Availability

Data are contained within the article and Supplementary Materials.
