# Supplementary material for: JNK inhibitor and ferroptosis modulator as possible therapeutic modalities in Alzheimer disease (AD)
Source: Sci Rep. 2024 Oct 7;14:23293. doi: 10.1038/s41598-024-73596-1 (PMC11458622; doi:10.1038/s41598-024-73596-1)
Supplement: Supplementary file 1 — Supplementary Material 1 [file 41598_2024_73596_MOESM1_ESM.pdf]

Supplementary Material

JNK Inhibitor and Ferroptosis Modulator as Possible Therapeutic Modalities in Alzheimer disease (AD)

Running title

CPX-O and SP600125 for Alzheimer disease management

Sherin Zakaria [a], Nashwa Ibrahim [a*], Walied Abdo [b], Alaa El-Sisi [c]

[a] Department of Pharmacology and Toxicology, Faculty of Pharmacy, Kafrelsheikh University, 33516, Kafrelsheikh, Egypt.

[b] Department of Pathology, Faculty of Veterinary Medicine, Kafrelsheikh University, 33516 Kaferelsheikh, Egypt.

[c] Department of Pharmacology and Toxicology, Faculty of Pharmacy, Tanta University, 31512, Tanta, Egypt

*Corresponding authors:

Nashwa Ibrahim; Tel:+201090116023;

E-mail: nashwa.ebrahim@pharm.kfs.edu.eg

nashwaibrahim49@gmail.com

Walied Abdo; Tel:+201004323114;

E-mail: walid.eid@vet.kfs.edu.eg

E-mail addresses:

sherin_zakaria@pharm.kfs.edu.eg

nashwa.ebrahim@pharm.kfs.edu.eg

walid.eid@vet.kfs.edu.eg

alaa.elsayed2@pharm.tanta.edu.eg

Supplementary figures caption

Fig. S1 Histopathological findings of brain sections in pilot study of control and diseased (AD) groups. (**a**) Brain (Cornus Ammonis CA2 of the hippocampus) of control animal showing normal neuronal cells indicates large neuronal cells within the granular cell layer (arrow), (**b**) Brain (Cerebral cortex) of diseased animal showing encephalitis associated with focal gliosis (arrow), and (**c**)Brain (Cornu Ammonis CA2 of the hippocampus) of diseased animal showing severe degree of atrophic ischemic injury of the neuronal cells of the granular cell layer (arrows) H&E, X200, bar= 100 µm.

Fig. S2 Original western blot analysis in different studied groups.

Fig. S3 showing the cell death scenario in the ferroptotic pathway. Ferroptosis is primarily caused by excessive intracellular Fe^{2+}, which oxidizes cellular lipids through a reaction known as the Fenton reaction, in which Fe^{2+} reacts with H_2O_2 generating ROS with subsequent peroxidation of cellular lipids. Also depletion of the cysteine glutamate antiporter system (Xc-system)/redox glutathione/glutathione peroxidase 4 (GPX4) plays a critical role in ferroptosis. CPX-O is an iron chelator which can inhibit the Fenton reaction and its related events with subsequent inhibition of ferroptosis and retardation of AD progression. Aβ is a pathological hallmark of AD, but it is unclear which event occurred first: JNK activation or Aβ accumulation, and what serves as the initiator for the other. JNK activation also phosphorylates tau protein leading to the formation of NFTs with AD development. SP600125, a competitive JNK inhibitor, can retard AD progression and inhibit ferroptosis through reducing lipid ROS formation, increasing the ratio of GSH/GSSG, and restoring the activity of GPX4.

Figures

a b

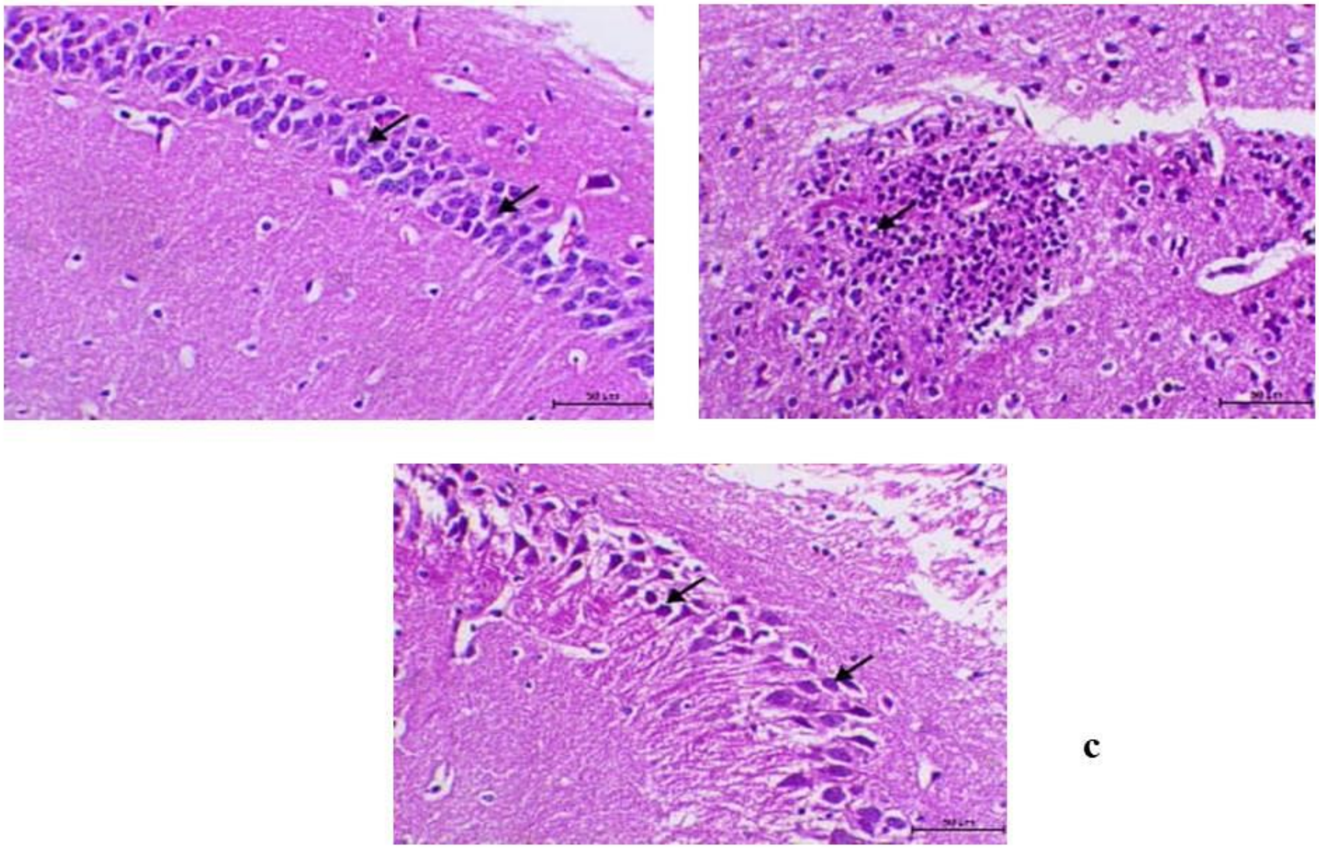

c

Fig. S1

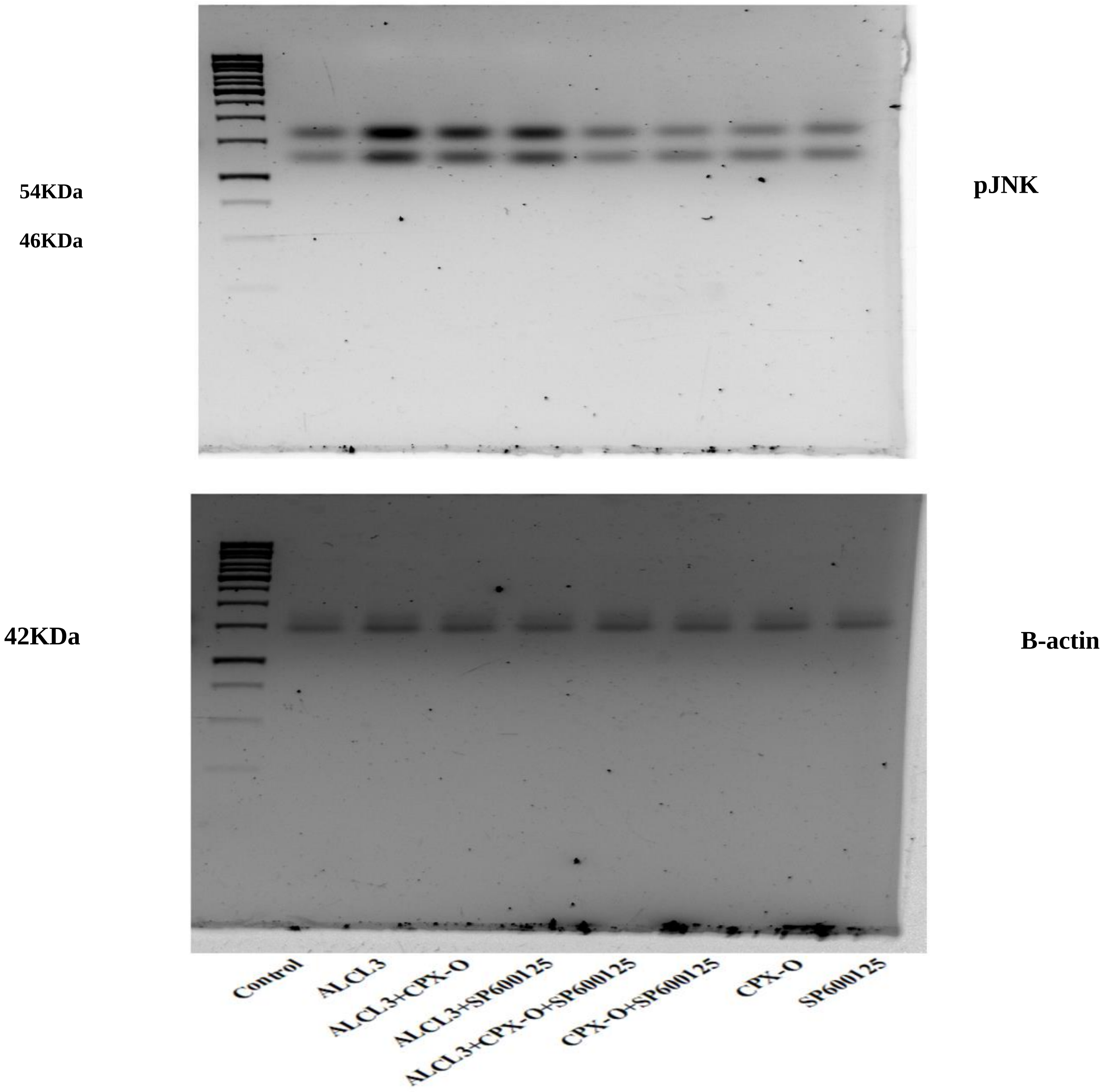
54KDa
46KDa
pJNK
42KDa
B-actin
Control
ALCL3
ALCL3+CPX-O
ALCL3+SP600125
ALCL3+CPX-O+SP600125
CPX-O+SP600125
CPX-O
SP600125

Fig. S2

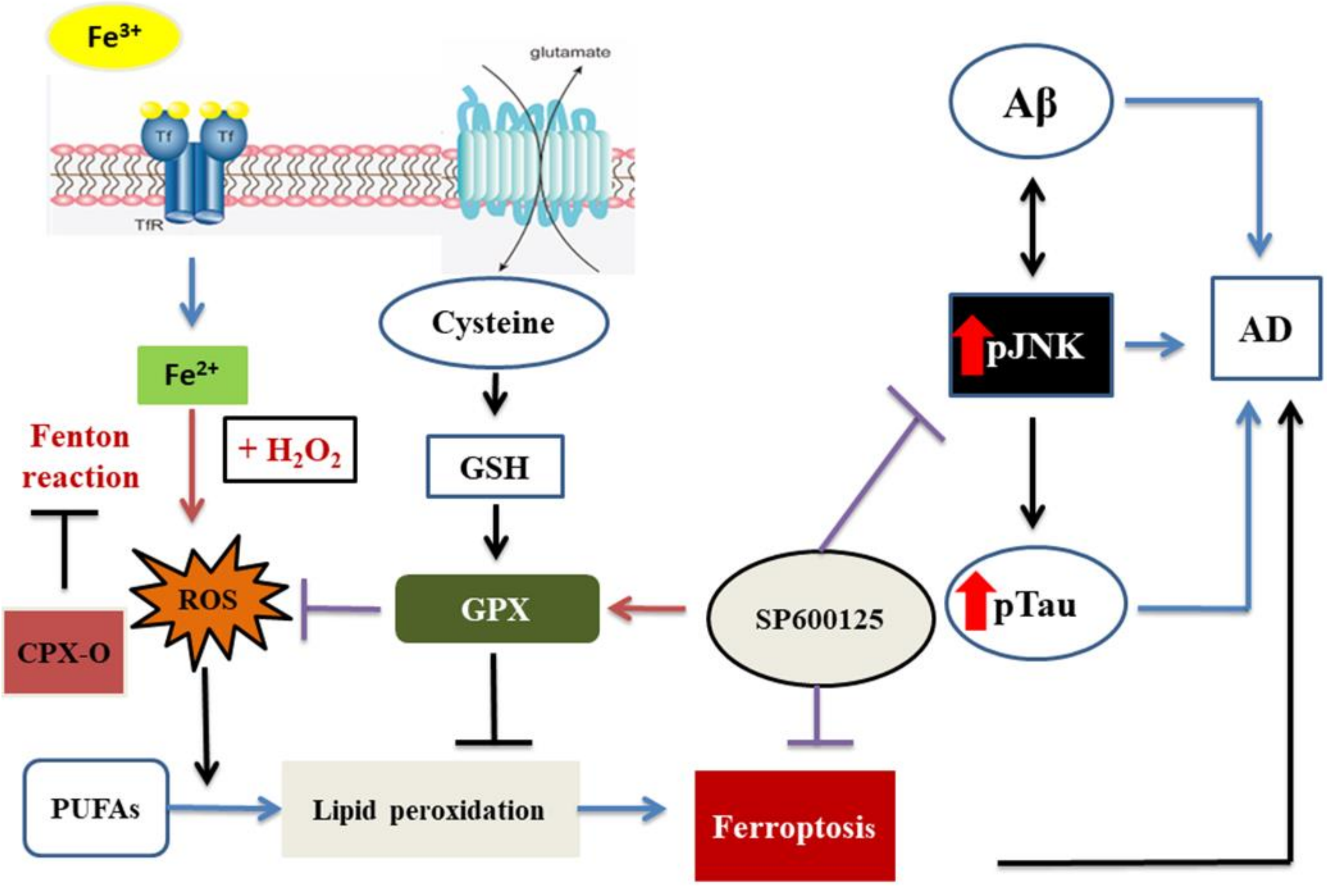

Fe3+
glutamate
Tf
Tf
TfR
Aβ
Cysteine
pJNK
AD
Fe2+
Fenton
reaction
+ H2O2
GSH
ROS
GPX
SP600125
pTau
CPX-O
PUFAs
Lipid peroxidation
Ferroptosis

Fig. S3
